# Transdiagnostic body dissatisfaction: comparing adolescents with anorexia nervosa and depression during body exposure

**DOI:** 10.1186/s13034-025-00939-9

**Published:** 2025-07-14

**Authors:** Valeska Stonawski, Lena Sasse, Laura Derks, Gunther H. Moll, Oliver Kratz, Tanja Legenbauer, Stefanie Horndasch

**Affiliations:** 1 Department of Child and Adolescent Mental Health, University Hospital Erlangen, Friedrich-Alexander University Erlangen-Nürnberg (FAU), Erlangen, Germany; 2 Psychotherapy and Psychosomatic, LWL University Hospital Hamm for Child and Adolescent Psychiatry, Ruhr University Bochum, Hamm, Germany; 3 Medical School, Department of Child and Adolescent Psychiatry and Psychotherapy, Bielefeld University, University Medical Center OWL, Protestant Hospital of Bethel Foundation, Bielefeld, Germany

**Keywords:** Anorexia nervosa, Body dissatisfaction, Depression, Adolescents, Body exposure, Anxiety, Disgust, Attentional bias

## Abstract

**Background:**

Body dissatisfaction (BD) is a risk factor for and a maintaining factor of Anorexia nervosa (AN). Furthermore, BD is associated with depressive symptoms. Body exposure (BE) was found to be an effective intervention for reducing BD. The current study aimed to investigate similarities and differences in BD between patients with AN and depressive symptoms and the efficacy of a computerized BE in those adolescents.

**Methods:**

We compared adolescents with AN (*n* = 36) to adolescents with depression and high body dissatisfaction (*n* = 21; DBD group). BD was assessed with questionnaires; valence ratings were obtained for different body parts. Emotion ratings and gaze patterns towards the own body were assessed during each session via rating scales and eye-tracking.

**Results:**

Satisfaction with several body parts increased and anxiety and disgust decreased throughout the intervention in both groups, with no significant differences between them. An attentional bias towards the three most unattractive body parts was found, expressed via longer viewing times; however, it was not modified by the BE intervention.

**Conclusions:**

The similarities between adolescents with AN and highly body dissatisfied ones with depression in terms of BD, emotional reactions to and gaze patterns on one’s own body suggest a transdiagnostic phenomenon of BD. The results suggest that a computer-based BE is an effective intervention for reducing BD.

**Trial registration:**

The study was pre-registered in the German Clinical Trials Register (Deutsches Register Klinischer Studien; DRKS), ID number DRKS00024675.

**Supplementary Information:**

The online version contains supplementary material available at 10.1186/s13034-025-00939-9.

## Introduction and aims

Body dissatisfaction (BD) is a common phenomenon among adolescents, with a prevalence ranging between 19 and 83% and higher scores in girls and overweight people [1, 2]. BD is related to body checking and body image avoidance [3], negative emotions and thoughts;, additionally, it is associated with an attentional bias (AB) in terms of e.g. looking longer at “ugly” than “beautiful” body parts [4–6]. Furthermore, BD has been shown as a risk factor for developing an eating disorder (ED) [7, 8].

In anorexia nervosa (AN), body image disturbance (BID) is a core feature: it involves distortions in perception, cognitions, emotions, and behaviors related to one’s body. Clinically, it is marked by negative emotions and thoughts related to body weight and shape, disturbed body perception, and pathologic behaviours. Thereby e.g., body image avoidance is typically seen [9], which reflects an overevaluation of weight and shape [3]. BID is considered an important maintaining factor of AN, a risk factor for a negative course of AN, and a negative predictor of inpatient treatment outcome [9, 10].

AN is, however, not the only clinical disorder associated with BD: Besides e.g. body dysmorphic disorder [11, 12] and social anxiety [13, 14], depression was found to be associated with BD, both in cross-sectional [15–17] and longitudinal [18] studies. This association was found to be mediated by self-esteem [15, 19]. Furthermore, high comorbidity rates were found for depression and EDs which can increase the severity of both disorders [20, 21]. Research on BD in adolescents with depression is less extensive than in AN, but equally important due to its prevalence and comorbidity.

Facing the high risk of BD in non-clinical and clinical samples, effective treatment options are important. Body exposure (BE) has been established as a central and effective intervention method in the treatment of BD [22–24]. In patients with AN, confronting their body activates BD and increases negative emotions and cognitions; furthermore, it raises an AB towards “unattractive” and/or shape- and weight-relevant body parts (e.g. [6, 25–27]). Repeated BE as a treatment intervention, however, reduces BD and ED psychopathology [28–30], and negative emotions in ED patients (“affective habituation”; [26, 31, 32]); preliminary data using BE in virtual reality hint to an AB reduction with more balanced gaze patterns towards the body [33, 34]. Similar BE effects were found for females with high BD: a decrease of BD, body image avoidance and shape concerns [4, 35, 36], a more positive rating of the most unattractive body part [36] and decreased negative emotions [35, 37]. Regarding the AB, however, a modification was only reported for healthy but not for body dissatisfied women [4, 38].

Despite the relevance of BD, patients with AN have not been compared to patients with depression regarding BD or the effects of BE. Doing this with adolescents for the first time, we hypothesized a transdiagnostic BD and expected differences in diagnosis-associated psychopathology, but not in BD and body image avoidance. In explorative analyses, we investigated the evaluation of body parts, adolescents’ affective reactions and gaze patterns towards their own body. We hypothesized that a four-session computer-based BE would be an effective intervention for both groups in terms of: (1) reducing BD and body image avoidance, (2) affective habituation, and (3) more balanced gaze patterns towards one’s own body.

## Methods

### Study design and procedure

The data was collected within the intervention module of the FRAnconian Longitudinal study of Anorexia Nervosa in Adolescents (FRALANA; study protocol see in [39]). The study was pre-registered in the German Clinical Trials Register (Deutsches Register Klinischer Studien; DRKS), ID number DRKS00024675. A standardized computer-based BE consisting of four sessions over a period of 2.5 weeks was evaluated in a controlled design for pre to post short-term effects. We compared adolescents diagnosed with AN (AN group) to depressed and highly body dissatisfied adolescents (DBD group) at baseline as well as throughout the BE intervention. The DBD group was surveyed at two locations, with *n* = 4 adolescents (19%) being recruited in a second study centre. All adolescents and their parents were treated in inpatient or day-clinic psychiatric setting due to their primary psychiatric diagnosis and gave informed consent before participation. The multimodal and interdisciplinary treatment followed the national treatment guidelines (S3 guideline) for the respective disorders and included evidence-based cognitive behavioral methods in individual and group setting with the involvement of caregivers. For AN, treatment involved realimentation and weight gain. After an adolescent agreed to participate in the study, no (additional) body image-related interventions were carried out during the study period in order to prevent any effect on the study results. An a priori power analysis for the primary outcome of the intervention module of the FRALANA study using standard values (*α* = 0.05, Power = 0.80) resulted in at least *n* = 23 participants per group. Ethical approval for the study was granted by the local ethics committees of the Medical Facultys of both study centres. The study was conducted in accordance with the Declaration of Helsinki.

**Fig. 1:**
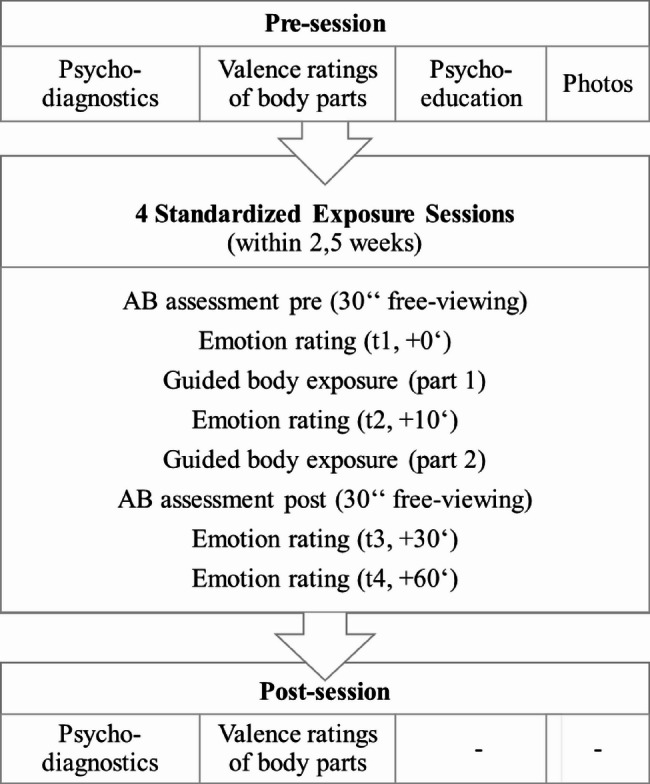
Study procedure. *Pre* before guided body exposure, *post* guided body exposure. Time within exposure session: t1 = 0’ = prior to start / anticipation, t2 = + 10’, t3 = + 30’ = end of exposure, t4 = + 60’ = 30’ after end / “recovery”

All adolescents participated in four exposure sessions (T1-T4) and completed a pre- and a post-session. Each session was standardized: The pre-session consisted of psychodiagnostics using questionnaires, valence ratings of body parts and a psychoeducation regarding the method of exposure; afterwards, standardized photos in frontal view and lateral view were taken with participants wearing standardized tight-fitting sportswear. At the post-session, psychodiagnostics and body part ratings were repeated and all participants received an expense allowance. During the four intervention sessions, the participants were placed comfortably at a 60 cm distance to a computer monitor and were confronted with their individual photos. They were guided through the BE sessions by a standardized audio file according to Vocks, Bauer [40], in which participants are guided to look at 15 different parts of their body in a fixed order from top to bottom. The audio recording was divided into two parts: the first focused after initial questions about the entire body on body parts from the head to the hands (hair, face, neck, shoulders, upper arms, forearms, hands; part 1: 10 minutes); the second addressed the torso to the legs (décolleté, chest, abdomen, waist, hips, thighs, lower legs, feet; part 2: 12 minutes). During the sessions, participants were instructed to neutrally describe the respective body parts, following prompts provided in the audio recording. Examples of such prompts included: ‘Are there differences between the left and right sides of your body?‘, ‘Now look at your mouth: what shape is it?’, and ‘How would you describe the transition from the waist to the hips?’ [40]. Emotion ratings were assessed at four time points: before, within, and two times after each session (t1 = start of intervention = 0’; t2 = + 10’; t3 = + 30’; t4 = + 60’). Gaze patterns towards one’s own body were analysed via eye-tracking during a 30-second free viewing task pre and post each session (t1, t3). The study procedure including all sessions and assessments are depicted in Fig. 1.

### Sample

Female adolescents between the ages of 10;0 and 17;11 years in inpatient or day-clinic psychiatric treatment were included in the study. For both groups, exclusion criteria covered acute psychotic symptoms, use of illegal substances, medication with sedating effects, chronic somatic diseases, intellectual disability (IQ < 85) and insufficient understanding of the German language. Moreover, all adolescents had to have reached a body weight > 10th body mass index (BMI) age percentile. This was done to avoid habituation at very low weight in participants, as recommended by, e.g. Griffen, Naumann [28] and Herpertz, Fichter [23].

For the AN group, participants had to be diagnosed with AN according to the ICD-10 criteria (F50.0 or F50.1) by an experienced child psychiatrist or psychologist. Inclusion criteria for the DBD group were a clinical diagnosis of depression based on the ICD-10 (F32 or F33), a score above the 75th percentile on the self-reported EDI-2 subscale “body dissatisfaction“, and a clinical judgment of high BD by an experienced child psychiatrist or psychologist. Exclusion criteria for the DBD group covered a comorbid diagnosis of an ED. For both groups, antidepressant or antipsychotic medication was allowed.

A total of 57 female adolescents (AN: *n* = 36; DBD: *n* = 21) between the ages of 11.6 and 17.8 years took part in the current study. *N* = 8 adolescents (*n* = 4 in each group) did not finish the complete intervention, lowering statistical power for especially the BE evaluation analyses: *n* = 6 were discharged from treatment before finishing the four sessions / post-session of the intervention (drop-out due to clinical procedure); *n* = 2 finished it earlier due to high emotional burden after BE sessions (drop-out due to study/intervention). Within the AN group, the majority had a diagnosis of a typical AN (restrictive type: *n* = 21; active type: *n* = 10) and *n* = 5 the diagnosis of an atypical AN. Comorbid diagnoses in the AN group were a depressive episode (*n* = 14), anxiety disorders (*n* = 11), a post-traumatic stress disorder (PTSD, *n* = 3), an adjustment or an obsessive-compulsive disorder (OCD; each *n* = 2), with *n* = 18 having more than one psychiatric diagnosis. Within a mean treatment duration of 14.8 weeks until study participation, the AN group gained an average of 7.5 kg (mean BMI at BE start: 18.0 kg/m^2^). Within the DBD group, all adolescents had a diagnosis of depressive disorder (*n* = 15 as main, *n* = 6 as comorbid diagnosis). Other main diagnoses were an anxiety disorder (*n* = 3), an OCD (*n* = 2) or PTSD (*n* = 1), other comorbidities were anxiety disorders (*n* = 10), PTSD (*n* = 4), an adjustment disorder, a borderline personality disorder, ADHD or a trichotillomania (each *n* = 1), with *n* = 12 having more than one psychiatric diagnosis. Within a mean treatment duration of 7.3 weeks until study participation, the DBD adolescents gained an average of 0.9 kg (mean BMI at BE start: 24.4 kg/m^2^). Overall *n* = 30 adolescents (AN: *n* = 18, DBD: *n* = 12) had more than one psychiatric diagnosis; both groups did not differ in the ratio of comorbid diagnoses (see Table 1).

### Measures

*Height and weight* were recorded at the pre- and post-session of the study and afterwards converted to *BMI*. *Psychometric data*: Psychopathology was assessed via reliable and validated questionnaires, with higher scores implicating higher psychopathology: BD-associated psychopathology was assessed with the Eating Disorder Inventory (EDI-2; [41], German validation: [42]), using the subscales ‘body dissatisfaction’ and ‘drive for thinness’. For the assessment of body avoidance behaviors the Body Image Avoidance Questionnaire ([43], BIAQ; German validation: [44]) was used. Depressive symptoms were assessed with the Beck Depression Inventory (BDI-II; [45], German validation: [46]). *Body part ratings*: To assess body (dis)satisfaction in relation to specific body parts, participants rated 17 body parts (hair, face, neck, décolleté, upper arms, chest, back, waist, underarms, abdomen, buttocks, hips, hands, thighs, knees, lower legs, feet) before and after the intervention on a 7-point Likert scale (-3 = “very negative”; 0 = “neutral”; +3 = “very positive”); furthermore, they rated their three most disliked body parts. These ratings were used to calculate (a) the mean (dis)satisfaction across all 17 body parts, (b) the mean (dis)satisfaction with the three most disliked body parts, and (c) the number of neutrally/positively rated body parts. *Emotion ratings*: Aversive emotions (anger, anxiety, disgust, sadness) were rated on an 11-point-Likert scale ranging from 0 (“not at all”) to 10 (“maximum imaginable”) at four time points within each session (see above). *Eye tracking*: Gaze patterns on the photos of one’s own body were recorded during a 1-minute free-viewing task (30’ each on the frontal and lateral photo, respectively) before and after each session by the Eyegaze Analysis System™ (Interactive Minds, Dresden, Germany), an infrared video-based binocular tracking system with a temporal resolution of 60 Hz. The three most disliked body parts were marked as regions of interest (ROI) for each adolescent. A fixation was counted if a participant’s gaze was directed to predefined areas of one degree for at least 100ms [47]. The total fixation time within the ROIs vs. the rest of the body were calculated separately for the frontal and lateral photo; the ratio of fixation time was then corrected for the size of the ROIs relative to the rest of the body (AB score). An AB score of 1 corresponds to a balanced gaze pattern on the entire body, an AB score > 1 is interpreted as an AB towards unattractive body parts.

### Statistical analyses

Descriptive group differences (AN vs. DBD) were tested by *t-* or *χ*^*2*^-tests. For testing the AB, a one-sample *t*-test was computed (null hypothesis: AB score = 1). For *t*-tests, Cohen’s *d* was used as the effect size measure [48]. Changes of the most disliked body part from pre to post intervention were analysed with a *χ*^*2*^-test. To test effects of BE regarding psychopathology and body part-specific dissatisfaction parameters, two-factorial mixed ANOVAs were calculated with the between-factor ‘group’ (AN vs. DBD) and the within-factor ‘time’ (pre vs. post intervention). To analyse the between and within course of emotion ratings (separately for each emotion), three-factorial mixed ANOVAs (between-factor ‘group’: AN vs. DBD; within-factor ‘session’: T1-T4; within-factor ‘time within session’: t1-t4) were run. For ANOVA results, effect sizes were computed as partial *η*^2^ (*η*_p_^2^) [48]. For the analysis of BE effects on gaze patterns, linear mixed models were calculated separately for the frontal and lateral photo: the abovementioned factors ‘group’ and ‘session’ and their interaction were used as fixed effects, the individual intercept as a random effect, and the AB change (AB score difference pre – post) as the dependent variable. Significant effects of ANOVAs and mixed models were further tested with post-hoc tests. Due to missing data and after quality control of eye tracking data, sample size differed between analyses. Relevant confounding factors (age, BMI, comorbidity, medication intake, treatment setting, treatment duration and weight change until start) were checked for two prerequisites: (1) groups differed significantly in the variable tested by *t*-tests, and (2) the variable was significantly and consistently associated with the outcome measures using Pearson correlation (*r*). These requirements were met for none of the tested variables, so no covariates were included. The level of significance was defined as *p* <.05; a correction for multiple testing with Bonferroni was applied for analyses regarding body part ratings and emotions.

## Results

### Sample group differences

In accordance with the group allocation by diagnoses, participants in the AN group had a significantly lower BMI and a higher weight gain from admission to the start of BE compared to the DBD group; DBD participants had more depressive symptoms. Both groups differed in treatment setting: while more AN participants were treated in an inpatient setting, more DBD participants were treated in a day-clinic. No group differences were found for age and medication intake. DBD participants of both study centres did not differ in age, BMI, ED, or depressive symptoms (all *p* >.05). Descriptive statistics and full results are shown in Table 1.

**Table 1:** Sample description and group differences

				AN (*n* = 32–36)	DBD (*n* = 17–21)	Group comparison AN vs. DBD		
Sample characteristics						*t / χ* ^2^	*p*	*d / r*
Age	Pre	in years	*M (SD)*	15.2 (1.6)	15.8 (1.5)	-1.41	0.165	0.39
BMI	Pre	in kg/m^2^	*M (SD)*	18.0 (1.1)	24.4 (5.8)	-4.99^a^	< 0.001*	1.77
BMI	Post	in kg/m^2^	*M (SD)*	18.4 (1.1)	23.7 (5.7)	-3.80^a^	< 0.001*	1.55
Weight change	Admission to pre	in kg	*M (SD)*	7.5 (3.9)	0.9 (2.0)	8.11^a^	< 0.001*	1.94
Depressive symptoms	Pre	BDI sum score	*M (SD)*	26.3 (14.9)	40.9 (9.1)	-4.58^a^	< 0.001*	1.11
Treatment characteristics								
Treatment setting	Inpatient		*n (%)*	27 (75%)	8 (38%)	7.62	0.006*	0.343
	Day-clinic		*n (%)*	9 (25%)	13 (62%)			
Treatment duration	Admission to pre	in weeks	*M (SD)*	14.8 (8.0)	7.3 (3.0)	5.00^a^	< 0.001*	1.14
Medication intake	Psychotropic drug intake		*n (%)*	14 (38%)	12 (57%)	1.78	0.182	0.174
Comorbidity	Yes (> 1 psychiatric diagnoses)		*n (%)*	18 (50%)	12 (57%)	0.27	0.602	0.069

*AN* Anorexia nervosa group, *DBD* depressed and body dissatisfied comparison group, *Pre* before intervention, *post* after intervention, *BDI* Beck Depression Inventory

^a^ corrected for unequal variances

*p <.05

### BD-associated psychopathology

Regarding BD-associated psychopathology in terms of BD, drive for thinness and body image avoidance assessed via questionnaires, we did not find any significant effect (*p* >.05) in the 2 × 2 mixed ANOVAs, as can be seen in Table 2A. Groups did not differ in BD-associated psychopathology and the BE intervention did not modify psychopathology in either group.

**Table 2:** Descriptives and ANOVA results of (A) BD-associated psychopathology and (B) body part ratings

		AN (*n* = 32)	DBD (*n* = 17)		ANOVA results		
		*M (SD)*	*M (SD)*	ME / IA	*F*	*p*	*η*_*p*_^*2*^
**(A) BD-associated psychopathology**							
Body Dissatisfaction	pre	39.0 (10.0)	42.9 (7.2)	Group	1.39	0.245	0.03
*EDI-2, subscale score*	post	38.3 (11.6)	41.2 (9.3)	Time	2.40	0.128	0.05
				Group x time	0.50	0.482	0.01
Drive for thinness	pre	30.9 (9.5)	34.2 (5.1)	Group	2.86	0.097	0.06
*EDI-2, subscale score*	post	29.0 (9.7)	33.7 (6.3)	Time	1.66	0.205	0.03
				Group x time	0.60	0.441	0.01
Body image avoidance	pre	36.0 (14.3)	40.9 (9.0)	Group	2.08	0.156	0.04
*BIAQ, total score*	post	35.2 (13.8)	40.9 (11.7)	Time	0.11	0.739	< 0.01
				Group x time	0.11	0.739	< 0.01
**(B) Body part ratings**							
Mean (dis)satisfaction across all body parts	pre	0.24 (1.17)	-0.43 (0.64)	group	2.99	0.090	0.06
	post	0.24 (1.17)	-0.13 (0.79)	time	3.65	0.062	0.07
				Group x time	3.83	0.056	0.08
Mean rating of the three most disliked body parts	pre	-1.14 (1.18)	-1.53 (0.44)	group	1.25	0.270	0.03
	post	-0.91 (1.28)	-1.20 (0.97)	time	5.79	0.020*	0.11
				Group x time	0.20	0.658	0.00
Number of neutrally or positively rated body parts	pre	4.66 (4.69)	1.53 (2.60)	group	5.26	0.026*	0.10
	post	5.22 (5.01)	2.59 (3.74)	time	4.53	0.039*	0.09
				Group x time	0.42	0.518	0.01

*AN* Anorexia nervosa group, *DBD* depressed and body dissatisfied comparison group, *pre* before intervention, *post* after intervention, *ME* main effect, *IA* interaction effect, *EDI-2* Eating Disorder Inventory-2, *BIAQ* Body Image Avoidance Questionnaire

Body part ratings: -3 = “very negative” to + 3 = “very positive”; 0 = “neutral”

*p <.05

### Body part ratings

Comparing AN with DBD participants regarding the *most disliked body part*, we identified no difference between both groups (*χ*^2^ = 10.0, *p* =.267): adolescents rated the abdomen (AN: 47%; DBD: 42%) and their thighs (AN: 36%; DBD: 19%) most frequently as the most disliked body part. After the BE intervention, 16 adolescents (AN: 28%; DBD: 41%) rated a different body part as the most disliked one compared to the pre-session, with no significant group difference (*χ*^2^ = 0.86, *p* =.354).

**Fig. 2:**
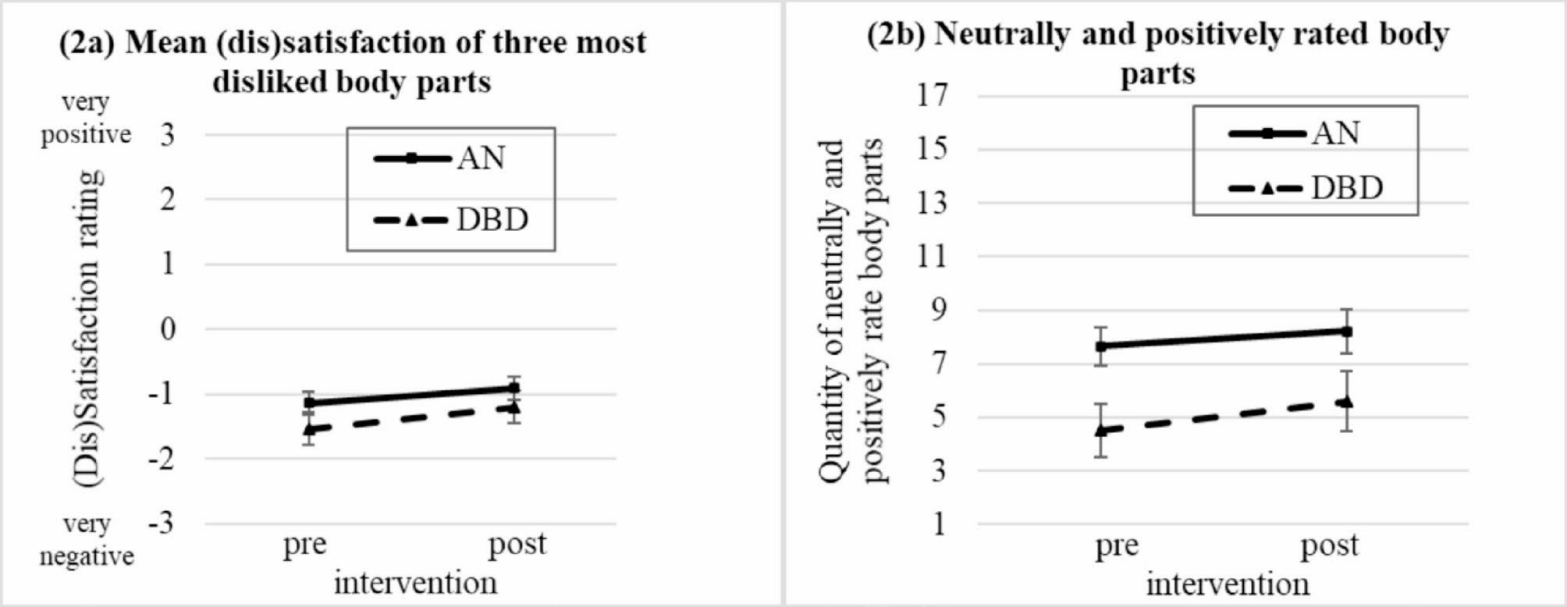
Changes in body part specific BD ratings from pre to post intervention. AN: Anorexia nervosa group, DBD: depressed and body dissatisfied comparison group. Ratings: -3 = “very negative” to + 3 = “very positive”; 0 = “neutral”. Pre: before intervention, post: after intervention

Regarding the initially *three most disliked body parts* within a 2 × 2 ANOVA, we found a significant increase of satisfaction from pre to post intervention in both groups (see Fig. 2a). Looking at the *number of neutrally/positively rated body parts*, we found significant main effects for group and time: the AN group rated more body parts as neutral or positive; moreover, both groups showed an increase of neutrally or positively rated body parts from pre to post intervention (see Fig. 2b). No significances were found regarding the mean dissatisfaction across all body parts. Full results are presented in Table 2B.

Regarding the 17 body parts, DBD participants were significantly more dissatisfied with their knees (main effect (ME) group: *F* = 12.25, *p* =.001, *η*_*p*_^*2*^ = 0.21) and feet (ME group: *F* = 11.73, *p* =.001, *η*_*p*_^*2*^ = 0.20) than AN participants. No other significant effects withstanding correction for multiple testing were found. Descriptives and results of all ANOVAs regarding the body parts are presented in supplementary material S1.

### Emotions

16 complete emotion ratings throughout the four exposure sessions were available for *n* = 26 AN and *n* = 10 DBD participants and analysed within a 2 × 4 × 4 mixed ANOVA correcting for multiple testing. Regarding *anxiety*, we identified significant main effects for session (*F* = 8.62, *p* <.001, *η*_*p*_^*2*^ =.46) and for time within session (*F* = 4.80, *p* =.008, *η*_*p*_^*2*^ =.32), both with large effect sizes. Demonstrating a between-session habituation effect, anxiety decreased continuously throughout all four sessions (T1 = 5.18, T2 = 4.18, T3 = 3.71, T4 = 3.17; post-hoc: *p* = <.001 −.053). Within each session, anxiety started to decrease continuously after 10’ into the BE (t2 = 4.34, t3 = 4.06, t4 = 3.72; post-hoc: *p* =.004 −.086).

**Fig. 3:**
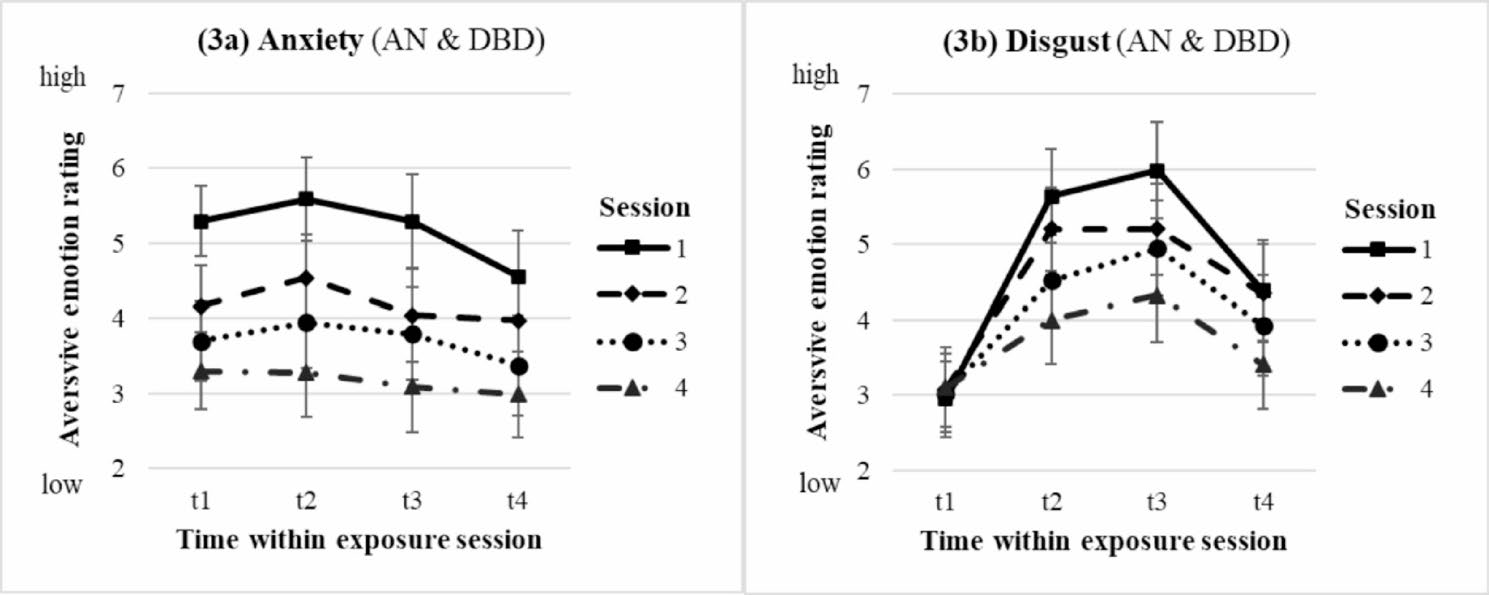
Emotion ratings within and throughout the BE intervention sessions. *AN* Anorexia nervosa group, *DBD* depressed and body dissatisfied comparison group. Time within exposure session: t1 = 0’ = prior to start / anticipation, t2 = + 10’, t3 = + 30’ = end of exposure, t4 = + 60’ = 30’ after end / “recovery”. Session 1–4 = four exposure sessions within 2.5 weeks. Data is presented as mean +/- SEM

Regarding *disgust*, we identified a session x time within session interaction effect (*F* = 4.54, *p* =.001, *η*_*p*_^*2*^ = 0.61). The intensity of the reactivity pattern in terms of an increase during exposure and a decrease after it decreased throughout all sessions indicating disgust habituation. No group difference was found for anxiety or disgust, so profiles of both emotions are shown together for the groups in Fig. 3 (3a: anxiety; 3b: disgust).

Regarding *anger* and *sadness*, we identified significant three-factorial interaction effects (group x session x time within session; *anger*: *F* = 3.75, *p* =.004, *η*_*p*_^*2*^ = 0.58; *sadness*: *F* = 3.33, *p* =.008, *η*_*p*_^*2*^ = 0.54) with large effect sizes. Post-hoc ANOVAs, conducted separately for each group, showed no significances for the DBD group, but a significant main effect for time within session for the AN group (*anger*: *F* = 5.22, *p* =.007, *η*_*p*_^*2*^ = 0.42; *sadness*: *F* = 3.76, *p* =.025, *η*_*p*_^*2*^ = 0.33): within a single session, both emotions increased continuously from t1 to t3 (*anger*: t1 = 2.19, t2 = 3.21, t3 = 3.48; *p* = < 0.001 – 0.006; *sadness*: t1 = 4.02, t2 = 4.58, t3 = 4.71; *p* =.002 – 0.004), followed by a decrease 30 min afterwards (t4 < t3; *anger*: t4 = 3.00; *p* =.023; *sadness*: t4 = 4.24; *p* =.022). Moreover, a significant main effect for session (*F* = 8.51, *p* <.001, *η*_*p*_^*2*^ = 0.53) was found for sadness, showing decreasing ratings throughout the four sessions (T1 = 4.91, T2 = 4.53, T3 = 4.18, T4 = 3.92; T1 > T3/T4, T2 > T4, post-hoc: *p* = < 0.001 − 0.011). Complete results of ANOVAs regarding the emotion ratings are presented in the supplementary material S2.

### Gaze patterns

One-sample *t* tests identified a significant AB for the AN group (frontal: *M(SD)* = 1.76 (0.53); lateral: *M(SD)* = 1.85 (0.70); *t* = 6.88 / 5.82, both *p* <.001) and a statistical trend for an AB in the DBD group (frontal: *M(SD)* = 1.60 (0.84); lateral: *M(SD)* = 1.86 (1.46); *t* = 2.26 / 1.86, *p* =.050 / 0.100) with consistently large effect sizes. The AN group in particular, but also the DBD group, tended to look longer at the most aversive body parts than at the rest of the body. Both groups did not differ statistically regarding the AB (frontal: *t* = 0.57, *p* =.577, *d* = 0.26; lateral: *t* = -0.02, *p* =.987, *d* < 0.01).

**Fig. 4:**
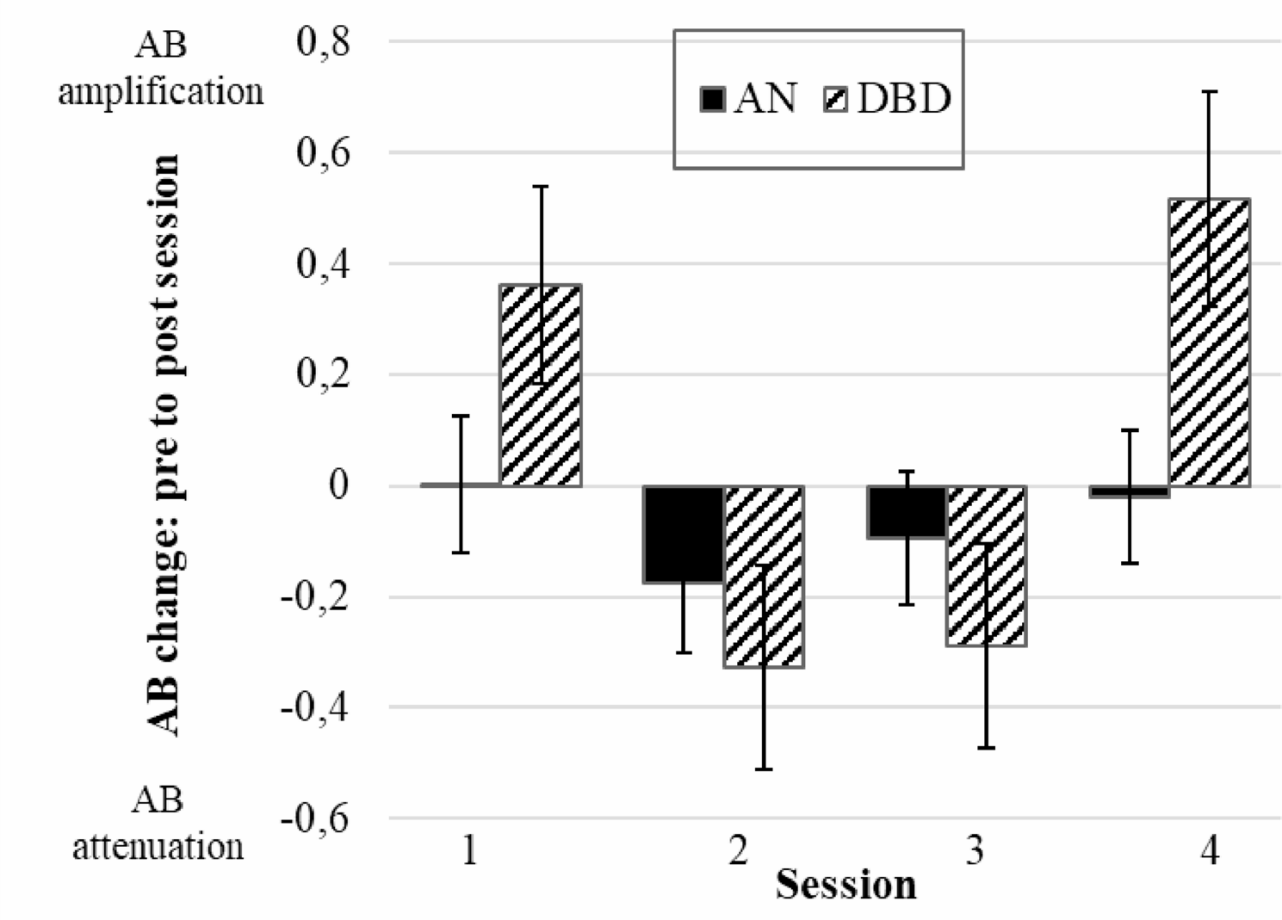
AB change from pre to post session separated by group. *AN* Anorexia nervosa group, *DBD* depressed and body dissatisfied comparison group. Session 1–4 = four exposure sessions. AB change from pre to post session: positive score (> 0) = AB amplification; negative score (< 0) = AB attenuation. Data is presented as mean +/- SEM

A linear mixed models (*n* = 21 AN and *n* = 10 DBD) analysis of the effects of the BE intervention on gaze patterns revealed a highly significant group x session interaction effect (*F* = 4.38, *p* =.005). While no differences were found in the AN group (*F* = 0.66, *p* =.577; AB differences: T1 = 0.00, T2 = -0.18, T3 = -0.10, T4 = -0.02), the AB change was not unidirectional and differed across sessions in the DBD group (*F* = 8.89, *p* = < 0.001; AB differences: T1 = 0.36, T2 = -0.33, T3 = -0.29, T4 = 0.52). Thus, adolescents with AN did not “respond” to the intervention with regard to the AB, while DBD adolescents showed an AB amplification in the first and last session vs. an AB attenuation in the second and third session (post-hoc: T1/T4 > T2/T3: each *p* <.001), as presented in Fig. 4. Full descriptives and results are presented in supplementary material S3.

## Discussion

In the current study, adolescents with AN were compared to adolescents with depression and high BD. No group differences were found for BD, most negatively rated body parts and an AB towards disliked body parts. Furthermore, they did not differ regarding the effects of a four-session computer-based BE intervention in terms of a reduction of body part-specific dissatisfaction and an affective habituation. Groups differed regarding the AB “activation” during an exposure session, hinting to a more robust phenomenon in the AN group.

### Body dissatisfaction

Supporting our hypothesis of a transdiagnostic phenomenon of BD, we found no difference for BD, assessed with the EDI-2, between both groups, but a diagnosis-conform difference for depressive symptoms. This finding supports the high prevalence and relevance of BD in both disorders [9, 10, 15–17]. Furthermore, no group differences were found for other BD-associated psychopathology measures (drive for thinness, body image avoidance), underlining the high similarity of both groups. This might be a consequence or a coping strategy due to the adolescents’ high BD, which should be investigated further.

Adolescents rated the abdomen and thighs, relevant to shape and weight, as the most aversive body parts, consistent with previous studies [4–6]. The DBD adolescents rated all body parts more negatively than the AN group, which showed more differentiated ratings, especially for shape and weight-related areas like the abdomen and thighs, but not feet or knees. Because BD is a multifaceted construct, it is to be expected that subgroups will show differences in the components and it is yet still be appropriate to speak of a BID. The RDoC (Research Domain Criteria; [49]) perspective could help by – albeit following a transdiagnostic approach – providing the opportunity to differentiate between specific disorders within domains and constructs. Within the construct “Perception and Understanding of Self” in the Social Domain the self-concept in depression is characterized by global negative self-evaluations which are known to be broad and pervasive (e.g. [50, 51]); in contrast, in EDs like AN, a more domain-specific self-concept centers around the overvaluation of body shape/weight as central to self-worth. This could explain why the AN group rated more body parts as neutral or positive (body parts which are maybe not that indicative of a person’s weight like the face, hands or hair), while the DBD group shows consistently negative evaluations. Future research should explore the hypothesis of a global vs. more specific body dissatisfaction and its association with self-image and self-esteem.

Regarding the effects of the BE intervention, we found an improvement of BD pre to post intervention in terms of more satisfaction with the three initially most disliked body parts and more neutrally or positively rated body parts in both groups. These findings support our hypothesis and are in line with former studies showing a positive effect of BE on BD in patients with AN and highly body dissatisfied women [4, 28–30, 35, 36].

Changes in BD, as assessed by the rating scale for 17 body parts, were not reflected in the subjective report assessed via the EDI-2 questionnaire. This might be due to the short study period (2 weeks), a low sensitivity to small changes, or the high stability of BD even during or after longer-lasting ED specific treatment (e.g. [52]). Follow-up timepoints would be helpful to assess effects in the mid- and long-term as, for example, Jansen, Voorwinde [36] found that body image avoidance further decreased from post session to a 1-month-follow-up.

### Emotions

Matching results from former studies and in line with our hypothesis, we found an increase of aversive emotions during confrontation with one’s own body [26, 36, 53] as well as an affective habituation for anxiety and disgust throughout the BE intervention [35, 37]. Similar habituation profiles were found for sadness and anger, however, these occurred only in the AN group, which might be explained by a statistical effect of unequal (and small) sample size.

BE was found to elicit diverse emotions [28, 54]. Besides anxiety, which is a typical target of exposure interventions [29, 55], disgust is increasingly discussed as a relevant risk and maintaining factor for EDs and BD [56, 57]: for example, self-disgust is linked to a negative body image and BID [58, 59]; moreover, stronger disgust during a BE was associated with more ED symptoms [60]. The current finding of a disgust habituation suggests potential for modifying a maintaining factor of BD, warranting further investigation.

### Gaze patterns

We found an AB towards the most aversively rated body parts, compared to the rest of the body, without finding a group difference. This is in line with former studies showing an AB to “ugly”, “unattractive”, or shape and weight relevant body parts in patients with AN [4–6] and overweight women [61]. Body dissatisfied adolescents with AN and depression seem to show similar patterns in the way they look at their own bodies. Fitting in with the current finding of a transdiagnostic AB, Lukas, Nuding [62] found similarities between adolescents with AN and depression in other cognitive biases, hypothesising further transdiagnostic phenomena: an interpretation and memory bias was found in both groups irrespective of the content (disorder-related and non‐disorder‐related information). They argue that transdiagnostic factors, such as low self-esteem or BD, may be important in explaining these patterns across diagnoses [62].

We did not find the expected AB reduction after four BE sessions. This finding differs from previous studies, which reported an AB modification in AN patients and healthy adults through BE [34, 38], but no effect in highly body dissatisfied women [4]. This could be due to the short intervention of only four exposure sessions within a time interval of 2.5 weeks, which might be too short to affect robust cognitive biases as the AB and might limit longer-term changes.

However, we identified a group difference regarding the AB change within each session: In patients with AN, the AB remained stable across sessions, suggesting the AB to be a robust phenomenon in AN. In contrast, in the DBD group, the AB changed between the BE sessions in terms of an amplification or an attenuation, indicating greater modifiability. Again, the RDoC framework allows us to link attentional processes (usually studied under the Cognitive Systems domain) with self-relevant content (from Perception and Understanding of Self under the Social Domain). Similarly to the effects described above, while body-focused ABs are present in both AN and depression, in depression they may be less pervasive and intense, likely reflecting a broader range of self-relevant concerns rather than a body-centric self-concept [63]. In AN, due to the over-representation of body shape/weight in the self-concept, pervasive and stable ABs have been shown towards body-related cues. These might be more specific and less variable than the less specific ABs observed in depression. Furthermore, cognitive body-related schemas in EDs (or even adolescents with subclinical ED pathology: [64]) persist across contexts and time, unlike – at least partly – the more mood-dependent schema shifts often observed in depression [65]. This could be a further reason why adolescents with AN did not “respond” to the intervention with regard to the AB as much as adolescents with depression. Due to the small DBD sample and inconsistent changes, the current finding should be interpreted cautiously and validated in larger cohorts. Nevertheless, results suggest a more robust AB in AN compared to BD and depression, which should be followed-up.

While the present study found effects on the momentary evaluation of body parts and aversive emotions, no modification of the implicit AB was found. This could be due to the type of exposure and the instructions given: the patients were instructed to repeatedly describe their body parts as neutrally as possible and were not guided toward cognitive restructuring. This could explain the habituation process found in the form of reduced aversive emotions but the lack of effects on core cognitive schemas. In order to achieve cognitive change, it may be necessary to actively encourage patients to reevaluate their body parts, which, to the best of the authors’ knowledge, has not yet been investigated. The relevance of the BE instruction is underlined by initial studies with body-dissatisfied and healthy female participants that asked them to focus their attention on positively vs. negatively evaluated body parts [36] or to describe their entire body in positive vs. negative terms [66]; thereby, differential effects on various aspects of BD were observed dependent on the instruction. Further, explicit Attentional Bias Modification Training using virtual reality showed initial positive effects on AB in healthy young women and those with AN [33, 38]. So, in future studies BE should be further investigated with regard to differential effects on explicit and implicit measures of BD depending e.g. on the BE type and instruction.

### Limitations

 Group sample sizes were unequal and, for some analyses, small due to missing values or after quality control; some results should be interpreted as being preliminary and validated in larger studies. The BE intervention consisted of only four exposure sessions within 2.5 weeks, which might be too short to modify robust phenomena as the AB, to find changes in psychopathology assessed via less sensitive questionnaires or to reach longer-term changes; future studies should increase the number of sessions, use more state-sensitive assessment methods, and add a follow-up timepoint to gain insight in mid- or long-term effects of BE. Within the current study procedure participants were not diagnosed using a structured clinical interview, but diagnoses were adopted from the diagnostic process during clinical treatment. Instead of a validated clinical cut-off, a double criterion consisting of 75th EDI-2 percentile and clinical judgment was used to define high BD in the DBD group, which should be compared in future studies to prove the representativeness for clinically significant BD. Baseline data on symptomatology were not collected at clinical admission, so that previous changes in this regard could not be reflected in the current study. Finally, transdiagnostic constructs, as, for instance, self-image or self-esteem, were not assessed here and should be added to future studies.

## Conclusions

The present study revealed more similarities than differences between adolescents with AN and adolescents with depression and high BD in terms of BD, confrontation, and the effects of a computerized BE intervention, supporting the transdiagnostic BD hypothesis. Future research should further explore BD and its links to other transdiagnostic constructs to clarify its role in ED risk and maintenance, and to develop effective prevention and intervention strategies. Larger studies with more exposure sessions and follow-up measures might enhance the knowledge about and the effectiveness of BE interventions for body dissatisfied adolescents, regardless of diagnosis. Clinically, our results support integrating computerized BE interventions into psychotherapy, highlighting their cost-efficiency.

## Electronic supplementary material

Below is the link to the electronic supplementary material.

Supplementary Material 1

## Data Availability

The datasets used and/or analysed during the current study are available from the corresponding author on reasonable request.

## Abbreviations

AN: Anorexia nervosa
BD: Body dissatisfaction
BE: Body exposure
DBD: Depressed and highly body dissatisfied group
