# Supplementary material for: Transdiagnostic body dissatisfaction: comparing adolescents with anorexia nervosa and depression during body exposure
Source: Child Adolesc Psychiatry Ment Health. 2025 Jul 14;19:76. doi: 10.1186/s13034-025-00939-9 (PMC12257810; doi:10.1186/s13034-025-00939-9)
Supplement: Supplementary file 1 — Supplementary Material 1 [file 13034_2025_939_MOESM1_ESM.docx]

Supporting information to

**Transdiagnostic body dissatisfaction: Comparing adolescents with anorexia nervosa and depression during body exposure**

Valeska Stonawski, Lena Sasse, Laura Derks, Gunther H. Moll, Oliver Kratz, Tanja Legenbauer & Stefanie Horndasch

***Table S1.*** ***ANOVA results of single body part ratings*.**

|  |  | **AN**  (*n* = 32) | **DBD**  (*n* = 17) |  | **ANOVA results** | | |
| --- | --- | --- | --- | --- | --- | --- | --- |
|  |  | ***M (SD)*** | ***M (SD)*** | **ME / IA** | ***F*** | ***p*** | ***η_p_^2^*** |
| Hair | pre | 1.81 (1.38) | 1.53 (1.42) | group | 0.12 | .727 | <.01 |
|  | post | 1.41 (1.29) | 1.94 (1.09) | time | <0.01 | .987 | <.01 |
|  |  |  |  | group x time | 6.25 | .016 | .12 |
| Face | pre | 0.25 (1.52) | -0.47 (1.42) | group | 1.80 | .186 | .04 |
|  | post | 0.34 (1.58) | -0.06 (1.30) | time | 2.62 | .112 | .05 |
|  |  |  |  | group x time | 1.04 | .313 | .02 |
| Neck | pre | 1.19 (1.45) | 0.35 (1.41) | group | 1.05 | .312 | .02 |
|  | post | 0.69 (1.55) | 0.71 (1.21) | time | 0.20 | .661 | <.01 |
|  |  |  |  | group x time | 6.56 | .014 | .12 |
| Décolleté | pre | 0.53 (1.46) | 0.29 (1.36) | group | 0.08 | .785 | <.01 |
|  | post | 0.63 (1.54) | 0.65 (1.46) | time | 1.23 | .273 | .03 |
|  |  |  |  | group x time | 0.41 | .524 | .01 |
| Upper arms | pre | 0.22 (1.66) | -0.65 (1.17) | group | 3.55 | .066 | .07 |
|  | post | 0.25 (1.61) | -0.47 (1.07) | time | 0.54 | .465 | .01 |
|  |  |  |  | group x time | 0.27 | .609 | <.01 |
| Chest | pre | -0.53 (1.41) | -0.41 (1.46) | group | <0.01 | .968 | <.01 |
|  | post | -0.03 (1.40) | -0.12 (1.62) | time | 8.32 | .006 | .15 |
|  |  |  |  | group x time | 0.56 | .458 | .01 |
| Back | pre | 0.66 (1.73) | -0.47 (1.07) | group | 3.85 | .056 | .08 |
|  | post | 0.59 (1.41) | 0.12 (1.41) | time | 2.33 | .134 | .05 |
|  |  |  |  | group x time | 3.56 | .065 | .07 |
| Waist | pre | -0.28 (1.65) | -0.71 (1.57) | group | 0.65 | .426 | .01 |
|  | post | -0.09 (1.42) | -0.35 (1.50) | time | 2.28 | .137 | .05 |
|  |  |  |  | group x time | 0.21 | .646 | <.01 |
| Underarms | pre | 0.88 (1.52) | 0.06 (1.25) | group | 1.79 | .188 | .04 |
|  | post | 0.56 (1.68) | 0.29 (1.21) | time | 0.43 | .836 | <.01 |
|  |  |  |  | group x time | 2.18 | .147 | .04 |
| Abdomen | pre | -1.09 (1.42) | -1.65 (0.70) | group | 1.23 | .273 | .03 |
|  | post | -1.06 (1.39) | -1.29 (0.99) | time | 2.50 | .121 | .05 |
|  |  |  |  | group x time | 1.75 | .192 | .04 |
| Buttocks | pre | -0.19 (1.58) | -0.94 (1.44) | group | 2.00 | .164 | .04 |
|  | post | -0.16 (1.69) | -0.59 (1.23) | time | 1.00 | .322 | .02 |
|  |  |  |  | group x time | 0.70 | .406 | .02 |
| Hips | pre | -0.34 (1.38) | -1.18 (1.07) | group | 3.83 | .056 | .08 |
|  | post | -0.22 (1.58) | -0.88 (0.99) | time | 2.48 | .122 | .05 |
|  |  |  |  | group x time | 0.40 | .529 | .01 |
| Hands | pre | 0.72 (1.82) | 0.06 (1.30) | group | 1.56 | .217 | .03 |
|  | post | 0.81 (1.60) | 0.35 (1.32) | time | 1.45 | .235 | .03 |
|  |  |  |  | group x time | 0.39 | .537 | .01 |
| Thighs | pre | -1.16 (1.39) | -1.29 (1.11) | group | 0.01 | .917 | <.01 |
|  | post | -0.94 (1.50) | -0.88 (1.50) | time | 4.76 | .034 | .09 |
|  |  |  |  | group x time | 0.45 | .508 | .01 |
| Knees | pre | 0.84 (1.37) | -0.41 (1.00) | group | 12.25 | .001* | .21 |
|  | post | 0.66 (1.34) | -0.47 (1.07) | time | 0.59 | .447 | .01 |
|  |  |  |  | group x time | 0.16 | .690 | <.01 |
| Lower legs | pre | 0.03 (1.56) | -0.53 (1.07) | group | 1.60 | .212 | .03 |
|  | post | 0.06 (1.59) | -0.35 (1.00) | time | 0.36 | .554 | .01 |
|  |  |  |  | group x time | 0.17 | .679 | <.01 |
| Feet | pre | 0.63 (1.66) | -0.94 (1.20) | group | 11.73 | .001* | .20 |
|  | post | 0.59 (1.43) | -0.71 (1.31) | time | 0.64 | .428 | .01 |
|  |  |  |  | group x time | 1.09 | .302 | .02 |

*Notes*. AN: Anorexia nervosa group, DBD: depressed and body dissatisfied control group. Pre: before intervention, post: after intervention. Ratings: -3 = “very negative” to +3 = “very positive”; 0 = “neutral”. *significance after correcting for multiple testing.

***Table S2.* ANOVA results of emotions.**

|  | ***F*** | ***p*** | ***η_p_^2^*** |
| --- | --- | --- | --- |
| **Anxiety: 2x4x4 ANOVA** (AN: *n* = 26; DBD: *n* = 10) | | | |
| ME group | 0.12 | .730 | <.01 |
| ME session | 8.62 | <.001* | .46 |
| ME time within session | 4.80 | .008* | .32 |
| IA group x session | 2.20 | .109 | .18 |
| IA group x time within session | 2.29 | .098 | .19 |
| IA session x time within session | 0.57 | .809 | .18 |
| IA group x session x time within session | 0.62 | .765 | .19 |
| **Anger: 2x4x4 ANOVA** (AN: *n* = 26; DBD: *n* = 10) | | | |
| ME group | 0.01 | .933 | <.01 |
| ME session | 0.09 | .963 | .01 |
| ME time within session | 6.59 | .001* | .39 |
| IA group x session | 2.01 | .133 | .16 |
| IA group x time within session | 0.39 | .763 | .04 |
| IA session x time within session | 3.51 | .006* | .56 |
| IA group x session x time within session | 3.75 | .004* | .58 |
| **Disgust: 2x4x4 ANOVA** (AN: *n* = 26; DBD: *n* = 10) | | | |
| ME group | 0.65 | .425 | .02 |
| ME session | 7.98 | <.001* | .43 |
| ME time within session | 14.27 | <.001* | .57 |
| IA group x session | 0.44 | .727 | .04 |
| IA group x time within session | 0.34 | .794 | .03 |
| IA session x time within session | 4.53 | .001* | .61 |
| IA group x session x time within session | 1.02 | .452 | .26 |
| **Sadness: 2x4x4 ANOVA** (AN: *n* = 26; DBD: *n* = 10) | | | |
| ME group | 0.35 | .557 | .01 |
| ME session | 7.05 | .001* | .40 |
| ME time within session | 4.34 | .011* | .29 |
| IA group x session | 0.25 | .864 | .02 |
| IA group x time within session | 0.85 | .475 | .07 |
| IA session x time within session | 3.64 | .005* | .56 |
| IA group x session x time within session | 3.33 | .008** | .535 |

*Notes*. AN: Anorexia nervosa group, DBD: depressed and body dissatisfied control group. ME: main effect; IA: interaction effect. Between-factor: group (AN vs. DBD); within-factor: session (T1-T4); within-factor: time within session (pre vs. post). *significance after correcting for multiple testing.

***Table S3.* LMM results of gaze patterns.**

| ***FRONTAL VIEW*** |  |  |  |
| --- | --- | --- | --- |
| **Fixed effects** | ***F*** | ***df*** | ***p*** |
| Group | 0.73 | 1, 29.1 | .966 |
| Session | 8.50 | 3, 201.4 | <.001** |
| IA group x session | 1.56 | 3, 201.4 | .005** |
| **Random effects** | **Estimate** | ***SE*** | ***p*** |
| Residual | .370 | 0.04 | <.001** |
| Intercept (ID) | .131 | 0.05 | .007** |
| **ICC** | .261 |  |  |
| ***LATERAL VIEW*** |  |  |  |
| **Fixed effects** | ***F*** | ***df*** | ***p*** |
| Group | 0.50 | 1, 25.6 | .487 |
| Session | 0.19 | 3, 197.5 | .903 |
| IA group x session | 1.86 | 3, 197.5 | .137 |
| **Random effects** | **Estimate** | ***SE*** | ***p*** |
| Residual | .891 | 0.09 | <.001** |
| Intercept (ID) | .042 | 0.05 | .435 |
| **ICC** | .045 |  |  |

Notes. Results of linear mixed models analyses of AB scores. Fixed factors: group (AN vs. DBD); session (T1-T4); time within session (pre vs. post). Random factor: participants’ intercept. ICC = intraclass correlation.
